# Validation of a roughness parameters for defining surface roughness of prosthetic polyethylene Pe-Lite liner

**DOI:** 10.1038/s41598-022-05173-3

**Published:** 2022-02-16

**Authors:** Nur Afiqah Hamzah, Nasrul Anuar Abd Razak, Mohd Sayuti Ab Karim, Siti Zuliana Salleh

**Affiliations:** 1 Department of Biomedical Engineering, Faculty of Engineering, Universiti Malaya, Kuala Lumpur, Malaysia; 2 Department of Mechanical Engineering, Faculty of Engineering, Universiti Malaya, Kuala Lumpur, Malaysia

**Keywords:** Biomedical engineering, Mechanical engineering

## Abstract

The Biosculptor's CNC milling machine, the Biomill, offered four different surfaces machined on positive models. This study aims to adopt the surface topography method in characterizing the four different surface roughness of polyethylene Pe-Lite liner as a product of the Biomill. Three surface parameters chosen were the arithmetic average (Ra), root mean square roughness (Rq), and ten-point height (Rz). The surface parameters were used to define the four different surfaces (STANDARD, FINE, COARSE, and FAST) and then compared with the same liner material from a conventionally fabricated socket. The Ra values of the conventional liner, 8.43 μm, were determined to be in-between the Ra values of STANDARD and FAST surfaces which were 8.33 μm and 8.58 μm respectively. STANDARD surface required 43.2 min to be carved while FAST surface took almost only a third of the time compared to STANDARD surface (conventional socket takes 2–3 days). The results of this study would be one of the guidelines to the prosthetists using the Biosculptor in socket fabrication to produce sockets according to the suitable surface to cater to different requirements and levels of activity of each amputee.

## Introduction

Computer-aided design and Computer-aided manufacturing (CAD/CAM) technologies have progressed intensively in the medical technology field, such as dentistry and prosthetic production over the last decade^1,2^. This technology allowed modern manufacturers to produce the high quality and accuracy of a product with impressive precision at a high level of productivity. The linkage of CAD and CAM has allowed the manufacturer to overcome the shortcomings (e.g., the high cost and low productivity) presented by the conventional numerical control (NC) system. The system does this by using the same command of instruction throughout the process of manufacturing. As a result, manufacturers are able to produce a predictable and consistent product and alter design faster in the CAD system without reprogramming the CAM machines^3^. Another benefit of the CAD/CAM system is, it allows the creation of databases, therefore each change made on the design can be stored digitally and used as references^4,5^.

The successful link between CAD and CAM systems was extensively studied in recent years, especially in the field of orthotic and prosthetic (O&P)^6,7^. One of the essential factors in evaluating a CAD/CAM system is analysing the system’s machined products. The surface roughness of a machined product machined by a CAD/CAM system added an important design feature, which in turn, influence the product’s properties such as rate of friction, wear resistance and strength^8^. Thus, surface roughness parameters are the widely used indicator of product quality^9^. Surface roughness plays an important role in O&P, especially in the fabrication of transtibial prosthetic sockets. A desirable surface roughness property at the liner-residual limb interface can either increase or decrease friction, improve wear, and the surfaces may improve heat conductivity^10^. The component of the socket’s liner acts by maintaining an even pressure distribution and must be able to lessen impact forces. An excellent liner material should be able to clinically fit perfectly to the shape of the amputee’s residual limb by supporting all the bony features, volume, shape changes and distributing stressed points. Failure to do so can lead to a variety of clinical issues and skin problems such as blister, friction, cyst, dermatitis, and skin lesion^11–14^.

One of the common liner materials that have been in use since the 1950s is the dense closed-cell foam liner^15^. The most popular foam liner is the Pe-Lite liner system. It is made from medium-density polyethylene foam. The use of polyethylene is ubiquitous because it is used in a lot of everyday items. The physical properties of polyethylene can be determined by multiple combinations of densities and different commoners, making the material utilizable in a wide range of applications, including socket liners^16^. The material is durable and cheaper than the state-of-the-art elastomeric gel liners^17^. During the process of socket fabrication, the foam material undergoes a thermoforming process then is quickly fitted to follow the shape of the socket^18,19^. There are various patents and technologies developed to ensure a reliable prosthetic liner-skin interface material is available^20,21^. To cater to the needs of amputees, prosthetists, researchers and manufacturers have created a range of different liner materials with different chemical compositions, geometry and mechanical properties to suit amputees individually^22^. Besides, surface roughness analysis of the Pe-Lite liner would allow researchers to design a reliable socket with the desired surface roughness. In doing so, a standard procedure would maximize the application of the CAD/CAM system in socket fabrication while providing significant clinical results customized to each amputee.

The advancement of the CAD/CAM system allows users to select the level of surface roughness that may improve amputees' lifestyles^23^. Surprisingly, there is a lack of study on different surface roughness from milling processes at socket-residual limb interface. Therefore, this study is designed to identify the most suitable level of roughness offered by the Bioscupltor CAD/CAM system and comparable with the Pe-Lite liner used in traditional socket fabrication. This research adopted and validated the topographical method using the most common surface roughness parameters to identify four different surface roughness and their mechanical properties on Pe-Lite liners obtained by the Biosculptor CAD/CAM system.

## Material and methods

### Subject and residual limb shape acquisition

The data used in this study was collected from a 68-year-old female amputee with a transtibial (left) amputation due to a diabetic ulcer. The participant was recruited from the University Malaya Medical Centre (UMMC), and this study was conducted with the approval and permission of the National Medical Research Register Secretariat Ethics Committee under registration number of NMRR-16-2106-32880. The participant underwent thorough briefing sessions and was well informed on the consent related to the study. All methods were carried out in accordance with relevant guidelines and regulations under the guidance and supervision of a Certified Prosthetist and Orthotist (CPO) of the International Society of Prosthetics and Orthotics (ISPO) Category-2. The participant has been using a patellar tendon bearing socket (PTB) with Pe-Lite liner for the past two years. The amputee’s residual limb was scanned five times using Bioscupltor’s Bioscanner, an electromagnetic handheld scanner with a built-in camera located at 45° angle at each other^22^. The scanner ‘sweeps’ the amputee’s residual limb, and each sweep represented a scanned image of a part of the residual limb. Once the 3D image of the residual limb was obtained, A CAD software called Bioshape was used to rectify the 3D images before the creation of positive models. However, no modification or rectification process was done on the images as it was not required for this study. The CAD models were converted into *cut files*, a CNC compatible file format, which was then send to Biosculptor’s CNC mill machine, Biomill to cut the positive models. The process above was done multiple times, and the best representation of the amputee’s residual limb (i.e. scanned sample with lowest percentage difference against manual measurement) was selected to be machined by the Biomill machine. An observation was made by comparing the machined surfaces with a liner obtained from a conventionally made socket. The conventional socket was made using the plaster of Paris method according to the standard P&O procedure^24^.

### Biomill CNC milling system

Biomill CNC milling is a vertically oriented milling system that created the final 3D transtibial model or the positive models by carving a polyurethane foam. It is a 4.5-axis milling system (X, Y, Z and T-axis) with the capabilities to allow the user to select the cuffing resolutions whilst having the flexibility and accuracy to produce sharp cuttings and trim lines. The multi-axis system enables the machine to have better functionality than the 3-axis milling system and can offer a more complex operation. Each axis travelled differently during the milling process. The X and Y-axis are responsible for the horizontal movement, Z-axis involved in vertical, and the additional T-axis allowed movement on the vertical plane. Another advantage of the multiple axes configuration system is that it permits optimization of the cutting time. The system did this by using a high-speed spindle system, and the foam was cut according to the 3D model set by Bioshape CAD software. This was achieved because Biomill acquired a rapid transverse speed rate up to 900 inches per minute (IPM) or 0.381 m/s and speeds up to 21,000 min^−1^^25^.

Biomill has options of choosing different types of milling process. There were four modes of finishing and the differences were identified by the distance between the passes of the spindle as it cuts. As a result, different finishing has different degrees of roughness namely STANDARD, FINE, COARSE, and FAST (Table 1). For a FINE finish, the model rotated 1 degree between each vertical cut, STANDARD finish required the model to rotate 2 degrees between each spindle pass, 3 degrees rotation required for the FAST finish and 4 degrees to achieve the COARSE finish. Most of the positive models milled out by Biomill selected FINE or STANDARD as the final finishes. However, this study utilized all four different finishes and produced one positive models for each surface. All machined polyurethane surfaces were produced at the same spindle speed and executed with the same cutting tool (a custom 0.5 inch of diameter ball end milling cutting tool with 4 tooth-cutter was used), which resulted in cutting conditions as shown in Table 1. The time it took to execute each mode of printing depended on the number of passes, which increased in numbers for less rough surface. So once the type of finish was selected, the correct file to be cut was loaded to the Biomill processing system. The system then suggested a default plug or foam type for the milling process (BK-2 plug type). The plug fixture was then mounted onto the turntable with a mandrel fitted securely into the base of the polyurethane plug. The milling process started as soon as the spindle was enabled. Biosculptor adopted a staggered cutting option where the machine performed several undefined cuts until the required depth of the model was reached. This method was applied to prevent excessive tool bits, to prevent wear on the z axis of the spindle bearings assembly and ultimately to produce smoother surface. Once finished, the Pe-Lite polyethylene liners were then thermoformed over the positive models and wrapped tightly with cling films to ensure that the surface of the moulds were transferred directly to the Pe-Lite liners. These Pe-Lite liners were then used to test the surface roughness.

**Table 1:** Cutting conditions for each machined surface.

	Standard	Fine	Coarse	Fast
Spindle speed (min^−1^)	4	4	4	4
Feed rate (Inches per min)	150	200	200	200
Max tool depth (mm)	1.2	4	1.2	0.6
Speed rate (second/pass)	9.6	9.2	7	9
No. of passes	270	541	134	179
Time taken (min)	43.2	83	15.6	26.9

### Surface roughness test

**Figure 1:**
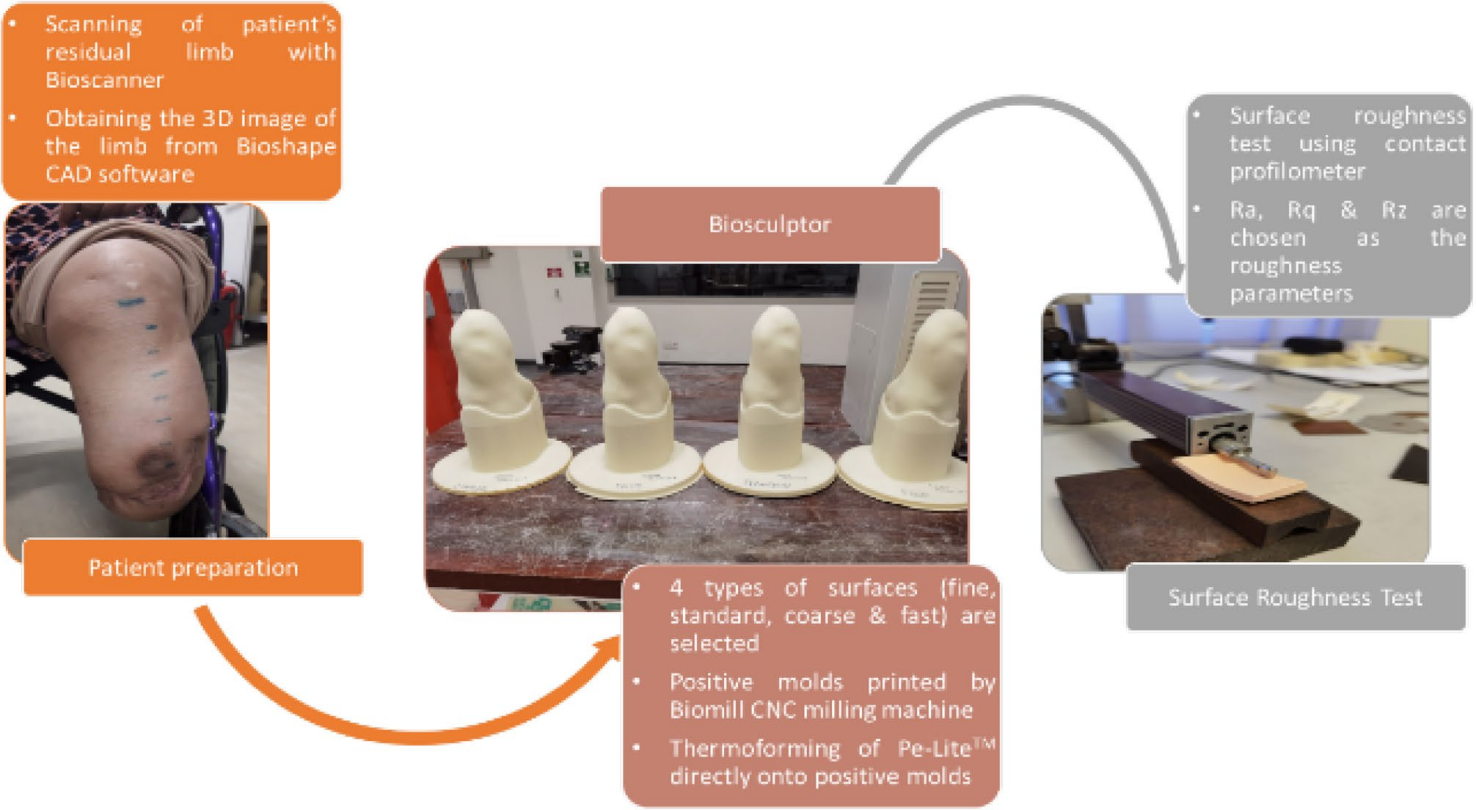
Diagram of the process of obtaining the four different types of surfaces of the Pe-Lite to be tested for surface roughness test.

For each surface, sample cutouts of about 2 cm × 6 cm were obtained as the benchmark samples. A common method to measure surface roughness is by using a profilometer^26^. The Pe-Lite samples were evaluated for surface roughness using a table-top contact profilometer device (Mitutoyo SurfTest SJ-210 series)^27,28^. The profilometer was fitted with a retractable probe with a diamond tip stylus. The stylus had a radius of 2 μm and equipped with a measuring force of 0.75 mN. Five to six benchmark samples were made for each surface, and twenty trials were carried out. The direction of the surface profiles measurements was perpendicular to the direction of the tool. The topographical analysis was carried using the profilometer which was connected to a communication software program that allowed an instant inspection to be recorded and automatically generated the 2D analysis graphs. From the twenty trials, twelve sets of surface roughness profiles were chosen to represent each surface. Three common roughness parameters, average surface roughness (Ra), root mean square roughness (Rq), and ten-point mean roughness (Rz) were selected. Figure 1 summarised the process from acquiring the shape of the amputee’s residual limb to the surface roughness test. Once the analysis was completed, a comparison of surface roughness analysis of the four surface samples against the surface of a Pe-Lite liner from a conventional socket was carried out.

To report the statistical measurement of validity, the twelve samples were used to provide the appropriate statistical measurement in the form of standard deviation (SD) and standard error of means (SEM). The mean values also reported for each surface parameter. The SEM values were calculated via Eq. (1) to estimate the accuracy and validity of the mean sample distribution, with n being the sample size. The calculation of SEM provides a measurement of the accuracy within a sample mean distribution using values of SD. A small standard error indicates a lesser spread of the sample's mean distribution thus, the sample's mean is more likely to be closer to the population mean^29,30^.

1 $$(SEM)=\frac{SD}{\surd n}$$

SD = standard deviation; n = sample size.

Lastly, to obtain the comparison data, the calculations were conducted for all three surface roughness parameters of the liners of machined sockets and the surface roughness of liner from conventional socket.

## Results and discussion

### CNC milling process and cutting conditions

Several parameters are crucial during the machining process, especially to improved surface quality or optimization. Parameters such as spindle speed, feed rate and tool depth/depth of cut are some of the example parameters which influence surface roughness properties. The process of producing the four positive models with different degrees of surface roughness were influenced by various factors such as the selected speed, feed rate, tool life, depth of cut and cutting temperature. Multiple studies looked at optimizing these various input factors to yield minimum total production cost^31,32^. Examination on the spindle speed resulted in a significant impact on the Ra, Rq and Rz values. A study on lowering the spindle speed caused variations on these surface roughness values^33^. Another study also indicated that spindle speed played a major factor in surface roughness properties^34^. As the spindle speed was the constant factor in this study, other parameters were automatically adjusted accordingly based on the surface roughness, selected as shown in Table 1.

Three conditions that were significantly varied between the surfaces were the max tool depth, number of passes and the time taken to execute each surface. The duration to execute the milling processes was directly correlated with the number of passes. A constant feed rate with increased max tool depth has resulted in an increased number of passes and a longer time to execute the process. At 200 inches per minute feed rate with 4 mm tool depth produced the FINE surface, meanwhile, at the same speed with 1.2 mm of tool depth, a COARSE surface was produced. It was also observed that an increment in feed rate caused an increment in the number of passes and time taken to execute a complete process. As a result, the FINE surface took the longest time of 83 min with 541 passes, while the COARSE surface took the shortest time of 15.6 min with only 134 passes. As a comparison, a high feed rate with a constant max tool depth has reduced the time taken to complete the process and produce a rougher surface. Therefore, the STANDARD surface was produced at 150 inches per minute with a max tool depth of 1.2 mm and with the same tool depth but a faster feed rate of 200 inches per minute, a rougher COARSE surface was produced. Much of the same outcome was observed with the speed rate. The COARSE surface required the lowest speed rate while the STANDARD surface with the fastest speed rate at 7 s/pass and 9.6 s/pass respectively. Nevertheless, the feed rate is shown here as the main factor in establishing the surface roughness of the machined products. Similarly, prior investigations too demonstrated that increasing the feed rate increased the surface roughness which resulted in low quality of surface finished product^35–38^.

The present study showed that the degrees and variation of surface roughness are varied as a product of various spindle speeds, feed rate and max tool depth. An earlier study has stated that good surface roughness can be obtained at high spindle speed, low feed rate and low tool depth^39^. The same study also emphasized that feed rate significantly affects surface roughness than other factors. These parameters can be seen in the FAST’s cutting conditions set by the Biomill software, which suggested that the FAST surface would have a suitable surface roughness for the transtibial socket. Another study also highlighted that several constraints sets would limit the optimization of the cutting conditions. Examples of such constraints are the limit on the cutting speed to ensure the safety of the machine while in use, the horsepower of the machine and the limit on the tool life^40^. The present approached reflected this with the feed rates which were maximized at 200 inches per minute have influenced the surface roughness^41^. Consequently, these cutting conditions resulted in different surface roughness which will bear different mechanical properties. Therefore, further analysis on the surface roughness is required to ensure that the surface roughness obtained from these pre-programmed cutting conditions would produce a suitable surface comparable with conventional socket liners and meet the clinical requirements desired.

### Surface roughness test

**Figure 2:**
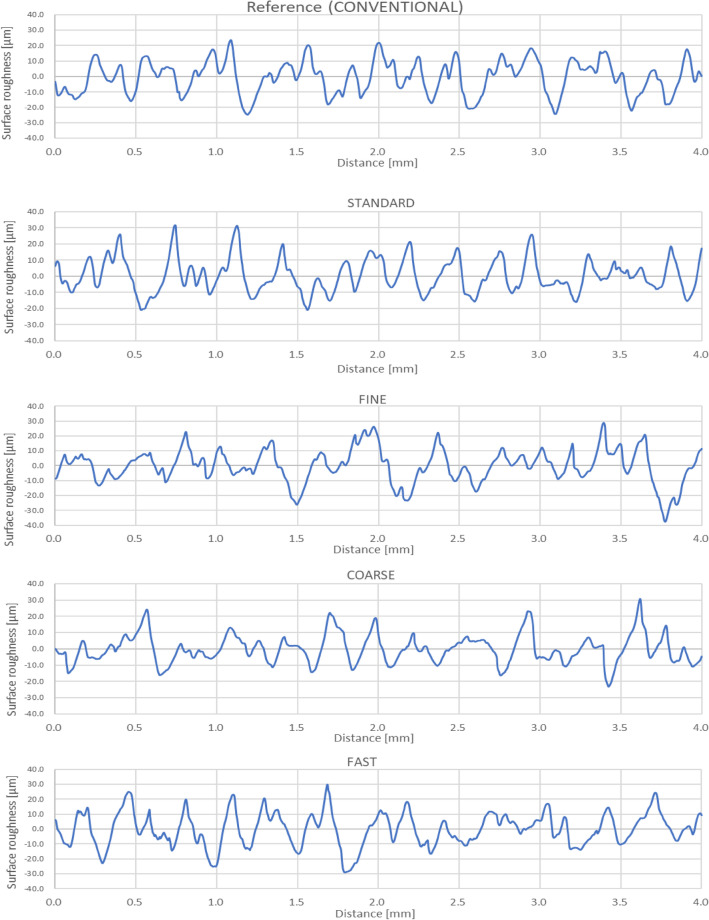
Cross-sectional profiles of the conventional Pe-Lite together with four of surfaces machined by Biomill CNC milling machine.

The cross-section lines of surface roughness profiles were generated for each sample and presented in Fig. 2. One example from each surface was chosen to be analysed, and the Pe-Lite liner from conventional fabrication was used as the reference sample. The communication program connected to the contact profilometer directly stored, analysed, and displayed all the results by subsequently plotting surface roughness (μm) versus distance data. The program also conveniently displayed the roughness profiles in a suitable height scale to optimise each result on a line graph. As a result, the peaks in the FINE surface as shown in (Fig. 2C appeared to be lesser than other rougher surfaces. The peaks are noticeably larger for the FINE surface and much smaller for the other three surfaces. Additionally, the results also showed that peaks of the standard STANDARD surface (Fig. 2B) are markedly similar to the conventional surface (Fig. 2A) in terms of its general irregularities, which indicated that the STANDARD surface may be comparable with the conventional surface. The coarse COARSE surfaces, as shown in Fig. 2D found to have a higher vertical roughness however, there is not much of a difference between COARSE, FAST, STANDARD and the conventional samples. The pattern of roughness irregularities shown in these 3 standards, COARSE, and FAST surfaces was almost analogous. The results also observed that the maximum peak and minimum depth for the conventional sample surface are much more uniformed than the machined surface. A range of maximum and minimum of ± 20 μm while the machined surfaces have a range of maximum and minimum varied between ± 20 μm and ± 35 μm.

There were three amplitude parameters studied to validate and analyse the surface roughness profiles. The arithmetic average height (Ra) is the most common parameter used for machined surface quality control. Ra indicates the average deviation of the surface irregularities from the mean line^42^. A high-speed cutting process would produce a smoother surface, thus a smaller Ra. As mentioned, the feed rate was highly influenced by Ra followed by tool depth and cutting speed which was evidently shown in this study. As shown in Table 2, the surface with the highest Ra was found to be the COARSE surface with Ra = 9.44 μm, meanwhile, the FINE surface had the lowest Ra with 7.98 μm. This indicated that a smoother surface was obtained for the FINE surface was related to the higher max tool depth and speed rate as compared to the COARSE surface at a constant feed rate. Meanwhile, relative to the conventional sample surface, the Ra value is slightly greater than STANDARD and FAST surfaces. The conventional sample acquired a Ra value of 8.43 μm while STANDARD and FAST have surface roughness measurements of 8.33 μm and 8.58 μm, respectively. The large difference between the FINE and STANDARD surfaces in terms of the time taken to complete the manufacturing is noticeable. Even though the feed rate for the FINE surface was higher than the STANDARD surface but due to the maximum tool depth to obtain a FINE surface is higher, thus the speed rate for the FINE surface is slightly slower than the STANDARD surface. This indicates that the influence of max tool depth is more significant than feed rate on the type of surface formation.

**Table 2:** Surface roughness analysis for 5 Pe-Lite surface samples.

	Ra				Rq				Rz			
	Mean	Median	SD	SEM	Mean	Median	SD	SEM	Mean	Median	SD	SEM
Conventional	8.43	8.27	0.65	0.19	10.19	9.95	0.72	0.22	40.54	39.48	2.85	0.86
STANDARD	8.33	8.30	0.23	0.07	10.13	10.01	0.35	0.11	40.84	40.53	2.28	0.69
FINE	7.98	8.02	0.72	0.22	9.87	9.75	0.80	0.24	40.79	40.75	3.32	1.00
COARSE	9.44	9.06	1.42	0.43	11.56	11.10	1.83	0.55	45.20	41.91	8.32	2.51
FAST	8.58	8.46	0.54	0.16	10.62	10.32	0.77	0.23	43.56	43.07	3.56	1.07

Results presented are averages (mean) of twelve measurements (Unit—µm).

*SD* Standard Deviation, *SEM* Standard Error of Means.

Based on the topographic method, the results are also intuitively expected for that root mean square roughness (Rq) values. The values for Rq which is also known as RMS Rq has followed a similar pattern with respect to Ra for both COARSE and FINE surfaces. Much like the Ra values, FINE surfaces have the lowest Rq values of 9.87 μm while COARSE have the highest Rq of 11.56 μm. Rq values for the conventional surface is also not much different from the STANDARD and FAST surfaces with a value of Rq of 10.19 μm, 10.13 μm, and 10.62 μm respectively. Rq is significant in describing the surface roughness as it depicts the standard deviation of the surface height distribution by statistical methods^42^. Rq is much more sensitive than Ra and provided surface roughness measurement at the microscopic level. Accordingly, Rq values are often used to compute the skew and kurtosis parameters of surface roughness and are one of the common parameters used in atomic force microscopy study in measuring nanoscale texture of surface roughness^43^. As Rq is the representation of the standard deviation of the surface heights, Rq values tend to be greater than Ra, as presented in the result, which agrees with reported results from a prior study^44^. Based on these results, the pre-programmed cutting condition has reflected the expected cutting conditions require to produce the required surface.

The ten-point mean roughness (Rz) of the COARSE surface, was found to be the highest with the mean value of 45.2 μm. Rz values give data on the presence of pores or other surface deformities resulting from the milling process and the depth of surface roughness profile irregularities^45^. Rz values are more sensitive to the presence of high peaks and deep valleys compared to Ra, which is reflected in the results which showed that the Rz for the FINE surface are the lowest with 40.79 μm while the COARSE surface has the highest Rz of 45.20 μm. The SD for most of the samples is in the range 0.2–2 except for Rz values. This may be due to the data is greater and more spread out due to the sensitivity of the parameter. As mentioned above, the calculation of SEM provides the measurement of the accuracy and high accuracy is obtained with smaller values of SEM. In this work, the majority of SEM for Ra, Rq and Rz values are less than 1 and only 20% of the data is above 1 which the smaller the values of SEM which indicates high accuracy measurements are obtained and the results are reliable for further investigation.

**Figure 3:**
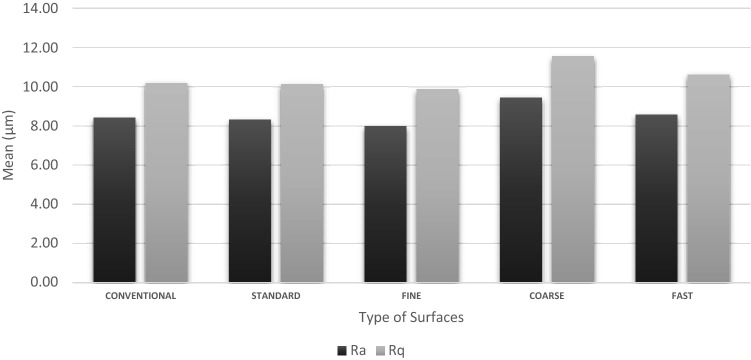
Line graph showing the Ra values of all surface samples (n = 12).

The differences between the conventional surface with the machined surfaces is shown in a bar graph depicted in Fig. 3. The graph highlighted the comparison of Ra and Rq values of each surface thus allowed direct comparison between the two values. Ra and Rq values for COARSE surface are distinctly much higher than other surfaces. Based on all the results, the conventional surface obtained Ra and Rq values that stretched between STANDARD and FAST surfaces which are clearly depicted in Fig. 3.

An overall overview of the results presented in current investigation, the topographical method and the statistical values of this study show that the Pe-Lite liner with STANDARD and FAST surfaces have the most comparable surface roughness characteristics when compared with the liner from conventional fabrication thus validate the significance of the current study. Hence it can be seen that it is more feasible to use CAD/CAM system to fabricate socket with surface roughness compared to the ones use for conventional fabrication.

## Conclusion

The four different of surface roughness machined by Biomill CNC milling machine were investigated to compare the surfaces with the liner used in conventional socket. To achieve the comparable surface roughness, the credibility of the surface obtained must be a reliable and suitable to achieve the clinical requirements. Surface roughness analysis is surely one of the methods that can be considered in achieving the goal. The cutting conditions presented by the system surely matched the degree of surface roughness selected. Further validation with topographical and statistical analysis, the following conclusions are drawn.

- Cutting conditions especially the feed rate, maximum depth of cut and the speed rate directly influenced the outcome of the surface. Finer surface requires greater number of passes than other rougher surfaces thus which in turn affected the time taken to finish the manufacturing process.
- The same pattern of results is measured for both Ra and Rq obtained. FINE surface has the lowest values of Ra and Rq while COARSE surface has the highest values for both. These are comparable with the conventional liner. Both Ra and Rq values of the conventional liner (Ra of 8.43 μm and Rq of 10.19 μm) stand between the STANDARD surface (Ra of 8.33 μm, Rq of 10.13 μm) and FAST surface (Ra of 8.58 μm, Rq of 10.62 μm).

Therefore, the result suggested that the machined surface from CAD/CAM machine is comparable to the liner’s surface from conventional fabrication. Having options of selecting the level of roughness on the socket-residual limb interface surely provided prosthetists with the additional tool to customise prosthetic sockets highly specific to the amputee’s daily needs. The designs offered by the Biosculptor CAD/CAM system can be studied further by analysing other roughness and waviness parameters and the effect of different degrees of roughness on friction, heat transfer and rate of liner’s degradation^10^. In conclusion, CAD/CAM system is a significantly valuable technology in modern O&P. Each component of the CAD/CAM system is not only practical, but the products are also evidently and proven to be comparable to the products of conventional fabrication.
